# Measuring the serum progesterone level on the day of transfer can be an additional tool to maximize ongoing pregnancies in single euploid frozen blastocyst transfers

**DOI:** 10.1186/s12958-019-0549-9

**Published:** 2019-11-29

**Authors:** Fazilet Kubra Boynukalin, Meral Gultomruk, Emre Turgut, Berfu Demir, Necati Findikli, Munevver Serdarogullari, Onder Coban, Zalihe Yarkiner, Mustafa Bahceci

**Affiliations:** 1 Bahceci Health Group, Hakki Yeten Cad. No: 11 Terrace Fulya, Fulya, Istanbul, Turkey; 2 Department of Statistics, Cyprus Science University, 99320 Dr. Fazil Kucuk Cad. Ozankoy, Kyrenia, Cyprus

**Keywords:** Serum progesterone, Frozen-warmed embryo transfer, Pregnancy, Euploidy, Embryo biopsy

## Abstract

**Background:**

Endometrial preparation with hormone replacement therapy (HRT) is the preferred regimen for clinicians due to the opportunity to schedule the day of embryo transfer and for patients due to the requirement of fewer visits for frozen-warmed embryo transfers (FET). The increasing number of FETs raises the question of the serum P levels required to optimize the pregnancy outcome on the embryo transfer day.

**Methods:**

This prospective cohort study includes patients who underwent single euploid FET. All patients received HRT with oestradiol valerate (EV) and 100 mg of intramuscular (IM) progesterone (P). FET was scheduled 117–120 h after the first IM administration of 100 mg P. The serum P level was analyzed 1 h before the embryo transfer (ET). In all cycles, only embryos that were biopsied on day 5 were utilized for FET. Next generation sequencing (NGS) was used for comprehensive chromosomal analysis.

**Results:**

Overall, the ongoing pregnancy rate (OPR) was 58.9% (99/168). Data were then categorized according to the presence (Group I; *n* = 99) or the absence (Group II; *n* = 69) of an ongoing pregnancy. No significant differences regarding, female age, body mass index (BMI), number of previous miscarriages, number of previous live birth, sperm concentration, number of oocytes retrieved, number of mature oocytes (MII), rate of fertilized oocytes with two pronuclei (2PN), trophectoderm score, inner cell mass (ICM) score, endometrial thickness (mm), oestrodiol (E_2_) and P levels prior to IM P administration were found between two groups. The P levels on the day of ET (ng/ml) were significantly higher in Group I (28 (5.6–76.4) vs 16.4 (7.4–60) *p* = 0.039). The P level on the day of ET was a predictor of a higher OPR (*p* < 0.001 OR: 1.033 95%CI [1.009–1.056]) after multivariate analysis. The ROC curve showed a significant predictive value of serum P levels on the day of ET for OPR, with an AUC (95%CI) = 0.716 (0.637–0.795). The optimal cut-off value for prediction of the OPR was a P level of 20.6 ng/ml (71.7% sensitivity, 56.5% specificity).

**Conclusions:**

The present study suggests a minimum threshold of the serum *P* value on the day of ET that needs to be reached in HRT cycles to optimize the clinical outcome. Individualization of the P dosage should be evaluated in further studies.

## Introduction

The number of frozen-warmed embryo transfer (FET) procedure has been increasing worldwide in the last decade. The main reasons underlying this circumstance are improvements in controlled ovarian hyperstimulation (COH) regimens, higher embryo survival rates after the implementation of vitrification in the laboratory and elective single embryo transfer (ET) policies. Freeze-all policies for the prevention of ovarian hyperstimulation syndrome and detrimental aspects of ovarian stimulation such as supraphysiologic oestrodiol (E_2_) levels and premature progesterone (P) elevation are factors that increase FET cycles. The best endometrial preparation protocol is still the subject of an ongoing debate [1–3]; however, hormone replacement therapy (HRT) is a more popular regimen owing to the opportunity to schedule the day of embryo transfer and its reduced monitoring requirements [4]. The best administration route of oestrodiol and P, the ideal dosage and duration, and the length of exposure to P before ET have not been well defined.

The increasing number of FET procedures raises the question of the serum P level that is required to optimize the pregnancy outcome because defining an optimal level may allow the individualization of FET in HRT. There is paucity of data on this topic, and most existing data are based on vaginal P administration [5–9]. A similar controversy is ongoing with respect to the intramuscular (IM) route. Data from two separate studies revealed lower pregnancy rates with low [10] and high [11] serum P levels on the day of ET. Recently, one prospective study on oocyte recipients [8] and one retrospective study conducted on euploid FET [7] showed a significant detrimental effect of a low serum P level on the day or the day before ET on pregnancy outcomes in patients receiving vaginal P administration. However, an optimal range on the day of ET was reported in a retrospective analysis [9].

Our aim in this prospective study is to determine whether an optimal P level exists for patients receiving IM administration on the day of FET for a successful outcome in cycles utilizing single euploid blastocysts.

## Materials and methods

### Design and setting

This prospective cohort study was performed at the Bahceci Health Group in Istanbul, Turkey, between March 1 and August 31, 2018. This study was approved by the Institutional Review Board with a reference number of 40. During this period, 328 euploid frozen-warmed blastocyst transfers were performed.

### Patient population

**Fig. 1:**
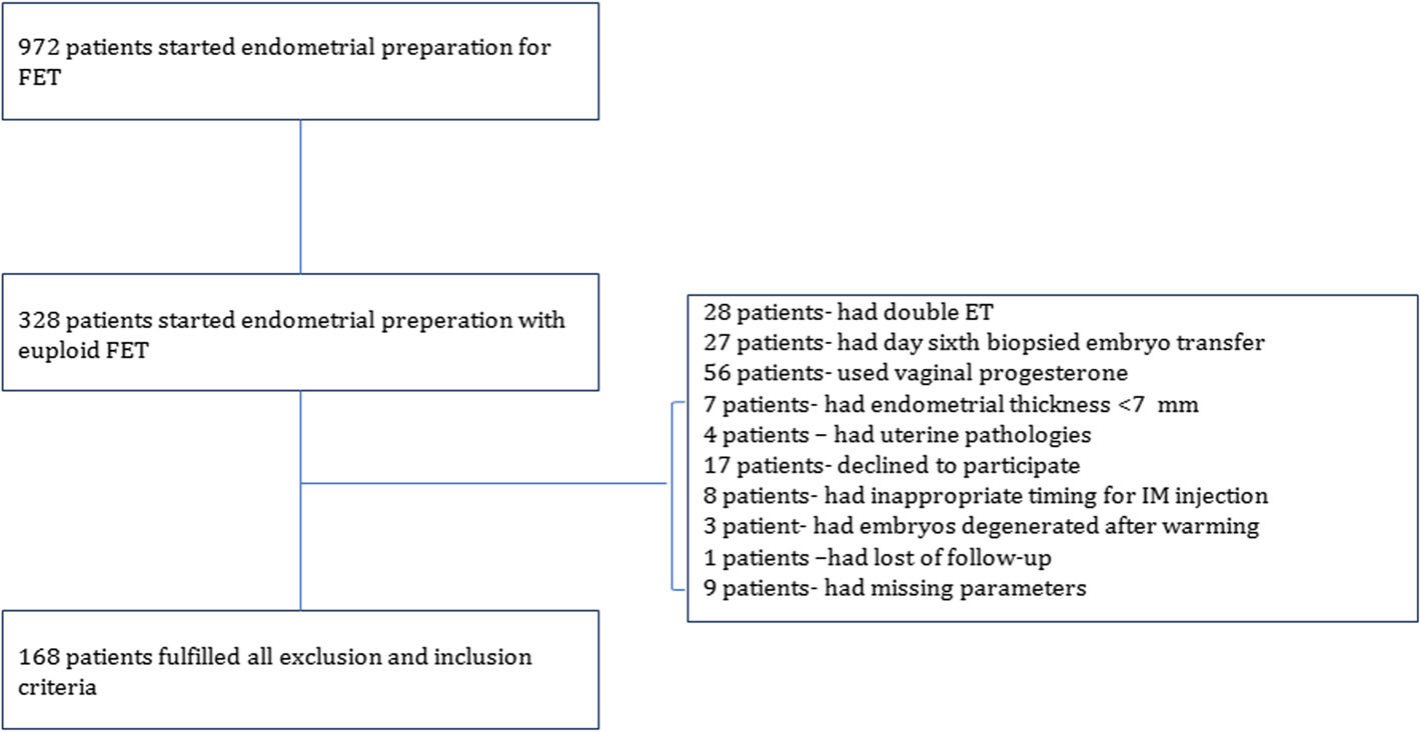
Flow chart of the study population

Between March 1 and August 31, 2018, 972 patients started endometrial preperation for FET were assessed for eligibility (Fig. 1). One hundred sixty-eight FET cycles utilizing euploid blastocyst were included. In all cycles, the embryos were artificially hatched on day 3 and biopsied on day 5 as hatching blastocysts. Only day 5 hatching blastocysts which are fully (100%) survived after warming procedure were included in this study. All patients underwent endometrial preparation with HRT. Patients with uterine diseases (e.g., fibroids, polyps, and previously diagnosed Müllerian abnormalities), the presence of hydrosalpinx or an endometrial thickness < 7 mm after HRT and embryos biopsied on day 6 were excluded.

In all cycles, COH, oocyte retrieval, denudation, intracytoplasmic sperm injection (ICSI), embryo culture, vitrification, and warming procedures were performed as described in detail previously by Serdarogullari and colleagues [12]. Moreover, embryo grading, trophectoderm biopsy, and embryo transfer were performed in accordance with standard operating procedures, as described below.

### Embryo morphology assessment and trophectoderm biopsy

The developmental characteristics of each individual embryo were recorded. The blastocyst morphology evaluation was performed at 114 h after ICSI according to the classification of Gardner and Schoolcraft [13].

Assisted hatching (AH) was applied to each embryo by creating a hole of approximately 20 μm using a laser pulse (OCTAX NaviLase, MTG, Germany) on day 3 of embryo development. After laser application on day 3, the embryos were transferred to fresh medium until the day of biopsy. Biopsy of each embryo was performed in 5 μl droplets of mHTF with Gentamicin (mHTF, Irvine Scientific, CA, USA) containing 10% SSS (Irvine Scientific, CA, USA). A trophectoderm biopsy was performed by the pulling method. On average, between five and eight cells were removed from the trophectoderm, and the extracted cells were placed in polymerase chain reaction tubes, and kept frozen at − 20 °C until PGT-A.

### Evaluation of viability after warming and extended culture

After the warming procedure was completed, the embryos were transferred to an equilibrated culture dish to assess the cryo-survival rate. Blastocyst grading was performed 2–3 h after the warming procedure. Viability after warming was quantified and classified based on the percentage of intact blastomeres (100%, ≥50, < 50, 0%) that were present in a blastocyst stage embryo and the blastocele re-expansion.

### Endometrial preparation and support

Endometrial preparation for FET involved HRT. Briefly, each woman was administered oral oestrodiol (Estrofem, Novo Nordisk, Istanbul, Turkey) in a step-up regimen (4 mg/day on days 1–4, 6 mg/day on days 5–8, and 8 mg/day on days 9–12). Transvaginal ultrasonography (TV-USG) (GE Ultrasound Korea Ltd., Korea, Model; Voluson S6) was performed on day 13 to measure endometrial thickness, and the cycle was cancelled if the endometrial thickness was < 7 mm. The serum P level was also measured, and the embryo transfer was cancelled if this concentration was > 1 ng/ml. Oral oestrodiol supplementation was continued at 8 mg/day, and IM administration of 100 mg of P (Progestan, Koçak Farma,Turkey) was started. Embryo transfer was performed on the 6th day of progesterone administration. Oral oestrodiol was continued until the 7th week, and IM P administration was continued until the 10th week of pregnancy.

### Serum analysis and hormone measurement

Blood samples were obtained to determine serum P on the sixth day of P administration, one hour prior to embryo transfer. Serum progesterone concentrations was measured by an electrochemiluminescence immunoassay (Cobas® Elecsys Progesterone III, Roche diagnostics GmbH, Germany). The intra-assay coefficient of variation was 2.4% and the inter-assay coefficient of variation was 3.9%. The sensitivity of the assay was 0.03 μg/l.

### Preimplantation genetic test for aneuploidy (PGT-A)

The NGS platform (Reproseq PGS Kit, Life-Thermofisher, USA) used in this study was previously validated and published elsewhere [14, 15]. Embryos were diagnosed as euploid, aneuploid or chaotic abnormal.

### Pregnancy outcome measurements

A human chorionic gonadotrophin (β-hCG) test was performed 12 days after embryo transfer. The test was considered positive if the β-hCG level was > 5 IU/l. Clinical pregnancy was defined as detection of an intrauterine gestational sac by TV-USG, and ongoing pregnancy was defined as a viable pregnancy detected by ultrasound examination at 16 weeks of gestation. Miscarriage was defined as loss of clinical pregnancy before gestational week 12.

### Data collection and analyses

The records of 168 patients from the same IVF center were examined to determine the cycle outcome. Thus, the clinical pregnancy, ongoing pregnancy and miscarriage rates were investigated. All statistical analyses were performed with the SPSS for Windows software package version 25 (SPSS, Chicago, USA). A *p-*value of ≤0.05 was considered to indicate statistical significance for all statistical tests.

First, the distributions of continuous parameters were evaluated using the Kolmogorov-Smirnov test to determine whether each variable followed a normal distribution. One hundred sixty-eight patients were then divided into two groups according to the presence or absence of an ongoing pregnancy. Because the continuous variables did not follow a normal distribution, they were reported as median (minimum-maximum) values, and a non-parametric independent median test was used to compare the values of the two groups.

To identify the approximate range of the statistically significant difference, the range of serum P levels on the day of ET was narrowed by examining quartiles; the 25th, 50th, and 75th percentiles (Q1, Q2, Q3 and Q4). Q1 included 0–25%, Q2 included 25–50%, Q3 included 50–75% and Q4 included 75–100%. Then, all possible two-way companions are performed between the quartile groups.

All categorical variables were compared among groups with a Chi-square test. To determine which factors affected the outcome of an ongoing pregnancy, a binary logistic regression analysis was performed with a forward stepwise conditional procedure. The variables included in the binary logistic regression model were female age, body mass index, sperm concentration, number of previous miscarriages, number of live birth, number of oocytes retrieved, fertilization rate, E_2_ levels on P administration day, P levels on P administration day, endometrium thickness and serum P levels on the day of ET. Only statistically significant factors were included in the final model and are reported in Table 3.

## Results

A total of 168 cycles of euploid blastocysts used for FET were evaluated. The β-hCG-positive rate, clinical pregnancy rate, OPR and miscarriage rate were 69.6% (117/168), 64.3% (108/168), 58.9% (99/168) and 8.3% (9/108), respectively. Data were then categorized according to the presence (Group I; *n* = 99) or absence (Group II; *n* = 69) of an ongoing pregnancy. Female age, BMI, sperm concentration, number of oocytes retrieved, number of miscarriages, number of previous live birth, number of mature oocytes (MII), the rate of fertilized oocytes with two pronuclei (2PN), and FET parameters such as endometrial thickness, E_2_ and P levels on the day of P administration were found to be similar between the groups. P levels on the day of the day of ET (ng/ml) were significantly higher in Group I (28 (5.6–76.4) vs 16.4 (7.4–60) *p* = 0.039) (Table 1). There was no difference in ICM and trophectoderm scores between the two groups.

**Table 1:** Characteristics of patients grouped according to the presence of ongoing pregnancy

Variable	Ongoing pregnancy (*n* = 99)	Non-ongoing pregnancy (*n* = 69)	*p*-value
Female age (years)	33 (23–44)	33 (26–42)	0.555
BMI (kg/m^2^)	23.9 (17.9–33.2)	24.6 (18.4–33.5)	0.683
Sperm concentration (million sperm/ml)	22 (0.4–75)	20 (0.1–60)	0.911
No. of oocytes retrieved	10 (3–35)	11 (1–40)	0.896
No. of MII oocytes	8 (2–28)	10 (1–33)	0.282
Fertilization rate (%)	87.5 (45.45–100)	83.3 (57.14–100)	0.318
E_2_ levels on P administration day (pg/ml)	260 (115–551)	286 (119–2018)	0.295
P levels on P administration day (ng/ml)	0.08 (0.05–0.90)	0.05 (0.01–0,.86)	0.332
Endometrial thickness (mm)	9 (7–12.3)	9,1 (7,.5–14)	0.494
P levels on the day of ET (ng/ml)	28 (5.6–76.4)	16.4 (7.4–60)	0.039*
No. of miscarriages	0 (0–4)	0 (0–5)	0.945
No. of previous live births	0 (0–1)	0 (0–1)	0.337
Trophectoderm Score			
A (%/n)	14.1 (14)	24.6 (17)	0.209
B (%/n)	34.3 (34)	27.5 (19)	
C (%/n)	51.5 (51)	47.8 (33)	
ICM Score			
A (%/n)	27.3 (27)	31.9 (22)	0.642
B (%/n)	63.6 (63)	56.5 (39)	
C (%/n)	9.1 (9)	11.6 (8)	

*statistically significant difference; *p* < 0.05

Values are given as the median (minimum-maximum) or number (percentage)

BMI, M2, 2PN, E_2_, and P denote body mass index, mature oocyte, 2 pronuclei, oestradiol, and progesterone, respectively

*p*-values were calculated with a Chi-square test and a non-parametric independent samples median test

The mean serum P level on the day of ET was 33.2 ± 23 ng/ml. Serum *P* values were divided into quartiles (Q). The serum P range for each quartile were Q1: < 13.6 ng/ml (*n* = 42), Q2: 13.6–24.3 ng/ml (*n* = 43), Q3: 24.4–53.2 ng/ml (*n* = 42), and Q4: > 53.2 ng/ml (*n* = 41). Table 2 shows the clinical outcome of patients in Q1, Q2, Q3 and Q4. Female age (33 (26–43), 32 (27–44), 34 (23–42) and 31 (28–44) respectively *p* = 0.86) and BMI (25.1 (20.7–29.6), 25.1 (17.9–32.8), 23.3 (18.4–33.5) and 22.3 (18.4–26.4) respectively *p* = 0.211) were similar among the 4 groups. Clinical pregnancy rates (15/42 (35.7%), 34/43 (79.1%), 25/42 (59.5%), and 34/41 (82.9) respectively, *p* < 0.001) and OPR (11/42 (26.2%), 32/43 (74.4%), 22/42 (52.4%) and 34/41 (82.9%) respectively, *p* < 0.001) were found to be significantly lower in Q1 group. Miscarriage rates (4/15 (26.7%), 2/34 (5.9%), 3/25 (12%) and 0/34 (0%) respectively, *p* = 0.015) were found to be higher in Q1 group.

**Table 2:** Clinical outcome according to serum P values (ng/ml) on the day of ET

Variable	Q1 (< 13.6 ng/ml) (*n* = 42)	Q2 (13.6–24.3 ng/ml) (*n* = 43)	Q3 (24.4–53.2 ng/ml) (*n* = 42)	Q4 (> 53.2 ng/ml) (*n* = 41)	*p*-value
Female age (years)	33 (26–43)	32 (27–44)	34.5 (23–42)	31 (28–44)	0.86
BMI (kg/m^2^)	25.1 (20.7–29.6)	25.1 (17.9–32.8)	23.3 (18.4–33.5)	22.3 (18.4–26.4)	0.211
Clinical Pregnancy Rate n (%)	15/42 (35.7)	34/43 (79.1)	25/42 (59.5)	34/41 (82.9)	< 0.001*
Ongoing Pregnancy Rate n (%)	11/42 (26.2)	32/43 (74.4)	22/42 (52.4)	34/41 (82.9)	< 0.001*
Miscarriage Rate n (%)	4/15 (26.7)	2/34 (5.9)	3/25 (12)	0/34 (0)	0.015*

*statistically significant difference; p < 0.05

Values are given as the median (minimum-maximum) or number (percentage). *p-*values were calculated with Chi-square test and non-parametric independent samples median test

*p < 0.05 for the following pairwise comparisons: Q1 vs Q2, Q1 vs Q3, Q1vs Q4

** p < 0.05 for the following pairwise comparisons: Q1 vs Q2, Q1 vs Q3, Q1vs Q4

***p < 0,05 for the following pairwise comparisons: Q1 vs Q2, Q1 vs Q4

When all of the parameters were assessed with binary logistic regression analysis to identify which covariates and factors effect the ongoing pregnancy outcomes, the serum P level on the day of ET (*p* < 0.001, OR: 1.033, 95% CI: 1.009–1.056) was the only significant variable (Table 3). The serum P levels on the day of ET was found to have a weak negative correlation with BMI (rho: − 0.284; *p* = 0.001) and had a positive correlation with the serum P levels on the day of P administration (rho: 0.224; *p* = 0.001) (Additional file 1: Table S1).

**Table 3:** Binary logistic regression analysis for ongoing pregnancy rate

Variable	B	OR	*p*-value	95% CI for OR
ET P4 levels	0.032	1.033	< 0.001*	[1.009–1.056]

*statistically significant difference; p < 0.05

**Fig. 2:**
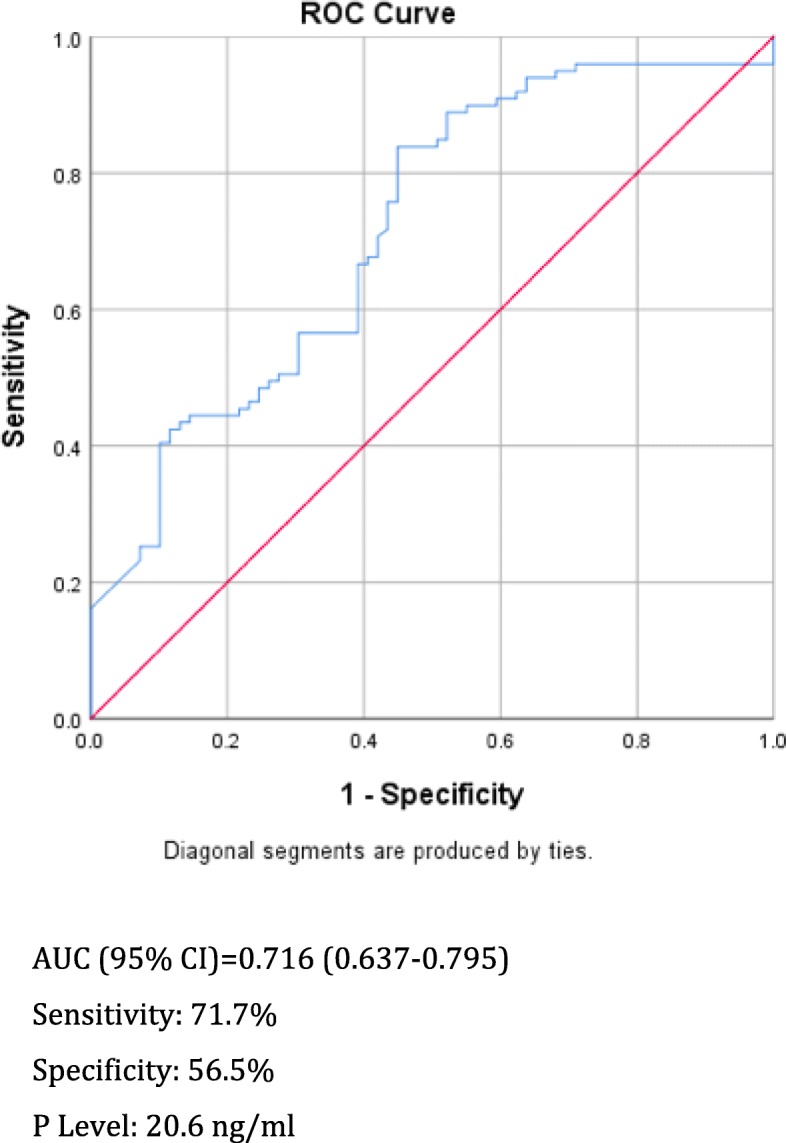
Receiver operating characteristic (ROC) curve for prediction of the OPR using serum P levels on the day of ET. AUC = area under the curve

The ROC curve showed a significant predictive value of the serum P level on the day of ET for the OPR, with an AUC (95%CI) of 0.716 (0.637–0.795). The optimal serum P threshold for which the sensitivity and specificity for the OPR was 20.6 ng/ml (71.7% sensitivity, 56.5% specificity) (Fig. 2). The OPR around this threshold was 41.8%(28/67) versus 70.3%(71/101) for serum *P* < 20.6 or ≥ 20.6 ng/ml, respectively (*p* < 0.001). Miscarriage rates were 15.2% (5/33) versus 5.3(4/75)%, respectively (*p* = 0.089).

## Discussion

In this prospective study, one of the inclusion criteria was the use of a single euploid, blastocyst biopsied on day 5 for FET. This approach enhanced the elimination of confounders such as female age, embryo quality, and COH protocols. The results of this study demonstrated that the P level on the day of ET utilizing a single euploid blastocyst was an independent prognostic factor for an ongoing pregnancy. Patients with serum P levels < 13.6 ng/ml prior to FET and who underwent endometrial preparation via HRT using IM administration of P had a significantly lower likelihood of an ongoing pregnancy. The ROC curve showed a statistically significant value, but the optimal threshold revealed modest specificity and sensitivity.

A receptive endometrium for the implantation of embryos can be achieved by exogenous administration of oestrodiol and P. The serum P concentrations achieved are higher with IM administration than with vaginal administration, whereas endometrial tissue P concentrations are higher after vaginal administration [16]. Regarding our clinical experience most patients opt for the vaginal route due to the self-administration advantage, ease of use, and reduced pain. However, in a recent, randomized, three-armed study, administration of 50 mg daily of IM P alone, twice daily vaginal administration of 200 mg P, and twice daily vaginal administration of 200 mg P plus IM administration of 50 mg P every 3rd day were compared in vitrified-warmed blastocyst transfers. The results showed that only the vaginal P arm had a significantly reduced pregnancy outcome [17].

Some factors may be able to alter P levels after vaginal administration, such as sexual intercourse, poor patient compliance and differences in vaginal absorption, distribution, and metabolism [18]. Low P levels were reported in more than one-third of patients who underwent daily vaginal administration of 600 mg micronized progesterone in a retrospective study [6]. Although increasing the vaginal P doshe to 1200 mg increased the serum P levels in most cases, it was still insufficient to improve the clinical outcome. These variations were also observed for IM administration [10, 11]. In our study, the serum P levels on the day of ET day exhibited a wide range. Although serum P levels on the day of ET were negatively correlated with BMI (rho: − 0.284 *p* = 0.001), this weak correlation is not sufficient to explain wide range of values. Personal metabolic variations should also be considered as a related mechanism. Inter-individual variations in serum P levels make it difficult to predict *P* values without monitoring the luteal phase. In addition, monthly or daily variations and their effect on clinical outcomes should also be evaluated.

Data regarding the optimal range of P values on the day of ET in cycles utilizing IM P administration are limited and conflicting [10, 11]. In 2014, Brady et al. evaluated the association between serum P levels on the day of ET and pregnancy rates in fresh donor IVF/ICSI cycles among day 3 transfers. They reported that serum P levels on the day of ET in fresh donor IVF/ICSI cycles were positively correlated with clinical pregnancy and live birth rates. The IM P dosage was not standard (50 or 100 mg/day) [10]. Additionally, regarding recipient P levels measured at ET, the P dose was increased by 50–100% if the level was < 20 ng/ml after ET. However, this was insufficient to rescue the pregnancy rates. Similar to our study, BMI was found to be related to P levels. Moreover, the study included donor cycles performed on patients with 1, 2 or 3 embryos transferred on day 3. The number and quality of embryos transferred were not reported in the comparison groups, which might cause a bias. In contrast to this study, Kofinas et al. retrospectively analyzed 213 single euploid ETs, and P levels > 20 ng/ml on the day of ET with a single euploid embryo were found to be associated with a decreased OPR and live birth rate [11]. In our study, the mean serum *P* value was 33.2 ng/ml, and almost 40% of the patients had a serum P value lower than 20 ng/ml. Kofinas et al. did not report the percentage or number of patients who had P levels > 20 ng/ml. However, the timing of P administration was changed two days later from morning to evening, which might affect the *P* values. The ET and serum sampling times were not stated. BMI was also not reported in the study, which could be an independent factor for the live birth rate and miscarriage rate after euploid embryo transfer [19]. In our study, BMI was found to be similar in the groups with and without ongoing pregnancy. Serum P levels on the day of ET were related to BMI, and after adjusting the variables, the only significant factor that could affect the OPR was the P levels on the day of ET. Although, the correlation between the BMI and serum P levels on the day of ET was weak, women with a higher BMI would likely benefit from higher doses of P from the start of administration. In the obese women, the thick subcutaneous tissue may negatively affect the optimal penetration of the lipophilic progesterone into the muscle, or their serum P levels may be lower due to a larger volume of distribution [10]. Further pharmacokinetic research is needed to define optimal dosing for these patients, to obtain serum P levels ≥13.6 ng/ml. However, according to the results of studies involving vaginal administration of P, increasing the dose does not proportionally increase the systemic and tissue P concentrations [18].

The effects of serum P concentrations on the development of endometrial histology and gene expression patterns were reported in a case–control experimental trial, including 46 healthy young females. Morphological delay was observed in the group supplemented with lower P concentrations. Higher P levels resulted in normal histology but aberrant gene expression [20]. This experimental trial supports the clinical study of Yovich et al., which reported that the likelihood of pregnancy in cryopreserved embryo transfer cycles under hormonal control is highly dependent on the circulating concentration of P, with an optimal P concentration of 70–99 nmol/l after vaginal administration [9]. Moreover, in animal studies, both low and high P concentrations were negatively associated with implantation [21]. Evidence based on one prospective and 4 retrospective studies using vaginal administration supports the negative impact of low serum P levels on pregnancy outcome, although the data do not support the previous finding that higher P levels have a detrimental effect, which is similar to the result of our study [5–8]. Future studies are warranted to analyze and monitor serum *P* values in standard luteal phase support.

P is essential for the implantation and maintenance of pregnancy; therefore any reduction in P levels or P resistance will likely be associated with changes in gene expression in the endometrium. Abnormal B-cell CLL/lymphoma 6 (BCL6) expression in the endometrium of infertile women was found to be associated with endometrial P resistance [22]. High BCL6 expression is a biomarker for endometrial inflammation and is associated with inflammatory proteins. In addition, oestrodiol and P are important components of immune reactions during implantation and pregnancy [23]. It could be speculated that the inflammatory pathway is regulated by certain serum and tissue P levels, and appropriate tissue activity of P should be achieved to allow for an adequate immunological environment to increase the likelihood of implantation and reduce pregnancy loss. Individualization of not only the dosage and but also the tissue activity of P may have a positive impact on the pregnancy outcome.

In artificial cycles, endometrial preparation is sustained both with vaginal and IM P administration. After the first dose, P levels increase rapidly in circulation and reach a steady state after 24 h, which makes monitoring of the luteal phase important [16]. A previous histological study showed that very low P levels are sufficient to induce histological endometrial maturation, but endometrial histological maturation is not a valid measure of the quality of luteal function or endometrial receptivity [24]. This result is supported by a clinical trial which showed that increasing the vaginal P administration dosage in FET cycles increased live birth rates and decreased abortion rates [25]. Additional studies are warranted to address the optimal route, dosage, and exposure time of P to individualize the luteal phase support in FET cycles.

The main limitation of the study is that only women with appropriate endometrial thickness and good quality euploid blastocysts were included. Extrapolation to different populations or to other doses of P via IM administration will require further validation. The study may have been underpowered to detect small differences.

## Conclusions

In conclusion, the results of our study showed that the P level on the day of ET is an independent prognostic factor for an ongoing pregnancy. The effectiveness of hormonal monitoring immediately before FET has not been proven to be beneficial. However, determining the threshold levels as well as the dose for the individualization of P treatment may improve the pregnancy outcome.

## Supplementary information

**Additional file 1: Table S1.** Relationship between serum P level on the day of ET and the basic and FET cycle characteristics of patients

## Data Availability

Available only on request.

## Abbreviations

AH: Assisted Hatching
AUC: Area Under The Curve
BCL6: B-cell CLL/lymphoma 6
COH: Controlled Ovarian Hyperstimulation
E_2_: Oestrodiol
ET: Elective Single Embryo Transfer
FET: Frozen-Warmed Embryo Transfer
HRT: Hormone Replacement Therapy
IM: Intramuscular
NGS: Next Generation Sequencing
P: Progesterone
PGT-A: Preimplantation Genetic Screening for Aneuploidy
PN: Pronuclei
TV-USG: Transvaginal Ultrasonography
β-hCG: Human Chorionic Gonadotrophin
