# Supplementary material for: Measuring the serum progesterone level on the day of transfer can be an additional tool to maximize ongoing pregnancies in single euploid frozen blastocyst transfers
Source: Reprod Biol Endocrinol. 2019 Nov 29;17:102. doi: 10.1186/s12958-019-0549-9 (PMC6884867; doi:10.1186/s12958-019-0549-9)
Supplement: Supplementary file 1 — Additional file 1: Table S1. Relationship between serum P level on the day of ET and the basic and FET cycle characteristics of patients [file 12958_2019_549_MOESM1_ESM.docx]

Additional file 1: **Table S1.** Relationship between serum P level on the day of ET and the basic and FET cycle characteristics of patients

|  | **P levels on the day of FET** | |
| --- | --- | --- |
|  | **rho** | ***p*** |
| **Age (years)** | -0.051 | 0.516 |
| **BMI (kg/m^2^)** | -0.284 | 0.001* |
| **E_2_ levels on P administration day (pg/ml)** | -0.002 | 0.980 |
| **P levels on P administration day (ng/ml)** | 0.225 | 0.005* |

*statistically significant difference; p<0.05
